# Soluble Endoglin as a Potential Biomarker of Nonalcoholic Steatohepatitis (NASH) Development, Participating in Aggravation of NASH-Related Changes in Mouse Liver

**DOI:** 10.3390/ijms21239021

**Published:** 2020-11-27

**Authors:** Ivone Cristina Igreja Sá, Katarina Tripska, Milos Hroch, Radomir Hyspler, Alena Ticha, Hana Lastuvkova, Jolana Schreiberova, Eva Dolezelova, Samira Eissazadeh, Barbora Vitverova, Iveta Najmanova, Martina Vasinova, Miguel Pericacho, Stanislav Micuda, Petr Nachtigal

**Affiliations:** 1 Department of Biological and Medical Sciences, Faculty of Pharmacy in Hradec Kralove, Charles University, 500 05 Hradec Kralove, Czech Republic; igrejasi@faf.cuni.cz (I.C.I.S.); tripskak@faf.cuni.cz (K.T.); eissazas@faf.cuni.cz (S.E.); vitverob@faf.cuni.cz (B.V.); najmanoi@faf.cuni.cz (I.N.); vasinovma@faf.cuni.cz (M.V.); 2 Department of Biochemistry, Faculty of Medicine in Hradec Kralove, Charles University, 500 03 Hradec Kralove, Czech Republic; HrochM@lfhk.cuni.cz; 3 Centrum for Research and Development University Hospital, Hradec Kralove, 500 03 Hradec Kralove, Czech Republic; RHyspler@lfhk.cuni.cz (R.H.); alena.ticha@fnhk.cz (A.T.); 4 Department of Pharmacology, Faculty of Medicine in Hradec Kralove, Charles University, 500 03 Hradec Kralove, Czech Republic; LastuvkovaH@lfhk.cuni.cz (H.L.); CermanovaJ@lfhk.cuni.cz (J.S.); eva.dolezelova@hotmail.cz (E.D.); 5 Biomedical Research Institute of Salamanca and Renal and Cardiovascular Physiopathology Unit, Department of Physiology and Pharmacology, University of Salamanca, 370 06 Salamanca, Spain; pericacho@usal.es

**Keywords:** endoglin, NASH, FFC diet, cholesterol, bile production, bile acids

## Abstract

Nonalcoholic steatohepatitis (NASH) is characterized by hepatic steatosis with inflammation and fibrosis. Membrane endoglin (Eng) expression is shown to participate in fibrosis, and plasma concentrations of soluble endoglin (sEng) are increased in patients with hypercholesterolemia and type 2 diabetes mellitus. We hypothesize that NASH increases both hepatic Eng expression and sEng in blood and that high levels of sEng modulate cholesterol and bile acid (BA) metabolism and affect NASH progression. Three-month-old transgenic male mice overexpressing human sEng and their wild type littermates are fed for six months with either a high-saturated fat, high-fructose high-cholesterol (FFC) diet or a chow diet. Evaluation of NASH, Liquid chromatography–mass spectrometry (LC/MS) analysis of BA, hepatic expression of Eng, inflammation, fibrosis markers, enzymes and transporters involved in hepatic cholesterol and BA metabolism are assessed using Real-Time Quantitative Reverse Transcription Polymerase Chain reaction (qRT-PCR) and Western blot. The FFC diet significantly increases mouse sEng levels and increases hepatic expression of Eng. High levels of human sEng results in increased hepatic deposition of cholesterol due to reduced conversion into BA, as well as redirects the metabolism of triglycerides (TAG) to its accumulation in the liver, via reduced TAG elimination by β-oxidation combined with reduced hepatic efflux. We propose that sEng might be a biomarker of NASH development, and the presence of high levels of sEng might support NASH aggravation by impairing the essential defensive mechanism protecting NASH liver against excessive TAG and cholesterol accumulation, suggesting the importance of high sEng levels in patients prone to develop NASH.

## 1. Introduction

Endoglin (Eng) is a transmembrane glycoprotein and co-receptor of transforming growth factor-β (Tgf-β) [1,2], which modulates Tgf-β signaling. Generally, there are two different forms of endoglin called membrane or tissue Eng, which are expressed by various cell types and soluble endoglin (sEng) circulating in plasma or cell culture medium [3]. Eng is expressed by endothelial cells, vascular smooth muscle cells [4], fibroblasts [5], hepatic stellate cells [6], and activated macrophages [7]. sEng is the N-terminal cleavage product of the extracellular domain of Eng formed by the activity of matrix metalloproteinases (MMP12 and MMP14) [8,9,10] that is released into circulation. Plasma concentrations of sEng are increased and can be used as a biomarker in patients with various cardiovascular and metabolic disorders such as familial hypercholesterolemia [11,12], arterial hypertension [13], preeclampsia [14], and type 2 diabetes mellitus [15]. 

Currently, knowledge regarding the regulation of Eng expression and sEng levels with respect to the liver functions and pathology is limited. It has been demonstrated that increased hepatic expression of Eng is mostly associated with the development of liver fibrosis [16,17,18]. Concurring, Eng expression has been shown to increase in activated hepatic stellate cells isolated from two in vivo models of liver fibrosis induced by bile duct ligation and carbon tetrachloride administration [19]. Additionally, significantly increased levels of sEng have been found in patients with hypercholesterolemia [12], type 2 diabetes mellitus [3], and in liver fibrosis induced by different causes (e.g., chronic hepatitis C, biliary atresia, cystic fibrosis-associated liver disease) [20,21]. These data suggest the importance of Eng and sEng in the process of liver fibrosis and liver alterations.

Nonalcoholic fatty liver disease (NAFLD) is a disorder characterized by excessive fat accumulation in the liver (hepatic steatosis) in the absence of excessive alcohol use history or other known liver diseases [22]. NAFLD is typically associated with obesity, insulin resistance, and type 2 diabetes mellitus, and it was previously recognized as the hepatic manifestation of metabolic syndrome [23]. However, recent studies show its mutual and bi-directional relationship with the metabolic syndrome, where NAFLD is also a potential precursor of metabolic syndrome and type 2 diabetes mellitus, and that the stage of fibrosis was a strong determinant of such a risk [24,25,26]. Nonalcoholic steatohepatitis (NASH) is the progressive form of NAFLD that results from the accumulation of triglycerides in hepatocytes (steatosis) associated with inflammation [23]. The presence of fibrosis in the liver suggests more severe progressive liver damage that can lead to liver cirrhosis, liver failure, hepatocellular carcinoma, and death [27]. It was demonstrated that the presence of advanced fibrosis in NAFLD patients results in greater risk for hepatic decompensation, hepatocellular carcinoma, and death when compared to patients with bridging fibrosis [28], for instance. Additionally, NAFLD patients with significant fibrosis are at higher risk with respect to the development of type 2 diabetes mellitus and arterial hypertension [29]. 

Knowledge regarding the relationship between NASH and Eng is currently limited to the observed increased intrahepatic expression of Eng in a liver biopsy of patients with NASH [30]. However, detailed experimental data on Eng and sEng effects on liver fibrosis and NASH progression are missing. 

Even though the underlying mechanism of NAFLD development and progression is poorly understood, it seems dependent on multiple hepatic insults [31,32]. One of them is an altered lipid metabolism that leads to increased fat accumulation, causing oxidative stress and, consequently, cellular damage [31]. Recent evidence has shown that the dysregulation of hepatic cholesterol metabolism led to elevated hepatic cholesterol levels in NASH and that hepatic accumulation of free cholesterol, together with free fatty acids, was critical for NASH progression [18,33]. Another underlying mechanism of NASH progression may be the bile acid (BA) toxicity [32]. A correlation between BA levels and NASH severity, together with dysregulation of BA signaling in NASH, was already described [31]. Additionally, the reduction of bile flow and BA accumulation induced hepatocyte cell death and liver injury [31].

During our previous study on mice fed with a chow diet, we showed that the presence of high levels of sEng resulted in an increased cholesterol content in the liver due to increased hepatic import of low-density lipoproteins (LDL) and total cholesterol in these mice. The presence of sEng also increased conversion of cholesterol into BA via upregulation of Cyp7a1 and, additionally, increased biliary elimination of BA coupled with choleretic activity [34]. 

Thus, in this study, we hypothesize that NASH induced by diet increases both Eng expression in the liver and the concentration of circulating sEng in blood. Moreover, we aim to explore whether increased levels of sEng modulate fatty acids, cholesterol, and BA metabolism and affect NASH induced by the experimental diet.

## 2. Results

### 2.1. FFC Diet Induce Metabolic Abnormalities and Liver Damage in Mice

**Figure 1:**
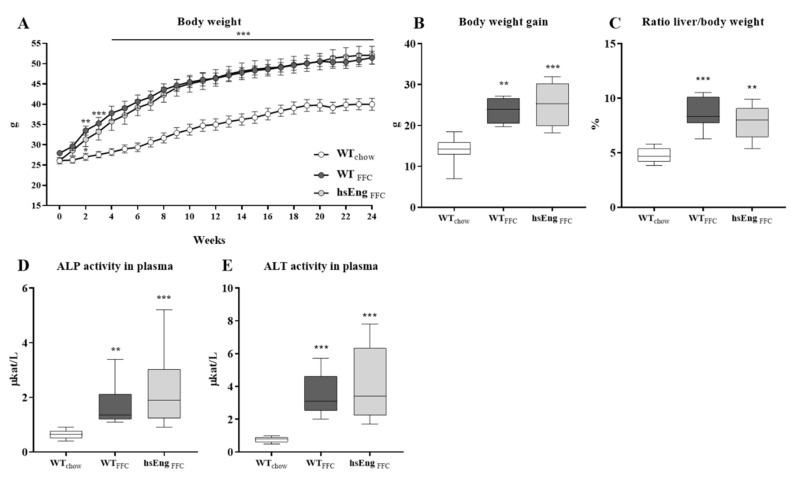
FFC diet and hsEng effects on body and liver weight as well as Alkaline phosphatase (ALP) and Alanine transaminase (ALT) activity. Increases in the bodyweight of each mouse were monitored at 1-week intervals throughout the 24 weeks of chow or FFC diet feeding (**A**). Bodyweight gain (**B**) and ratio liver/body weight (**C**) of mice after the feeding period. The activity of ALP (**D**) and ALT (**E**) in plasma. The data are presented as median with box and whiskers representing the interquartile range and 5th–95th percentiles (*n* = 8). * *p* < 0.05 ** *p* < 0.01, *** *p* < 0.001, using the Kruskal–Wallis test.

Mice fed with an high-saturated fat, high-fructose, and high-cholesterol (FFC) diet, as previously described [25,35,36], showed significantly increased body weight, from the 2nd week of feeding for wild type (WT) and 3rd week for high human soluble endoglin mice (hsEng) (Figure 1A), which resulted in obesity (significantly increased body weight gain (~29%)) at the end of the 24th week (Figure 1B) when compared to mice fed with a chow diet. Furthermore, mice fed with an FFC diet also showed a significantly increased ratio of liver weight to body weight (Figure 1C), which demonstrates hepatomegaly in these animals. Moreover, we detected significantly increased levels of alkaline phosphatase (ALP) (0.6 µkat/L–1.7 µkat/L) (Figure 1D) and alanine aminotransferase (ALT) (0.8 µkat/L–3.5 µkat/L) activity in plasma (Figure 1E), suggesting liver impairment induced by an FFC diet. 

Even though an FFC diet successfully induced metabolic abnormalities, the presence of high hsEng levels did not affect body weight gain during or after the feeding period (Figure 1A,B), the size of the liver ratio liver/body weight (Figure 1C), or the ALP and ALT activities in plasma (Figure 1D,E).

### 2.2. FFC Diet Increased Protein Expression of Liver Endoglin and Mouse Soluble Endoglin Levels in Plasma 

**Figure 2:**
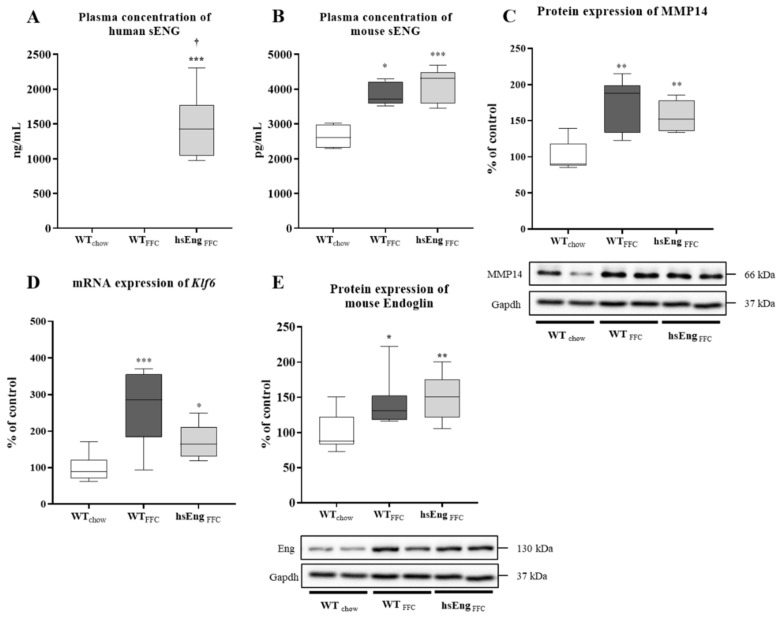
Human and mouse sEng levels in the blood, Eng, *Klf6*, and MMP14 expression in liver. Plasma levels of human sEng (**A**). Plasma levels of mouse sEng (**B**). Protein expression of MMP14 (**C**). mRNA expression of transcription factor *Klf6* (**D**). Protein expression of mouse Eng (**E**). The data are presented as median with box and whiskers representing the interquartile range and 5th–95th percentiles (*n* = 8). * *p* < 0.05, ** *p* < 0.01, *** *p* < 0.001, using the Kruskal–Wallis test; † *p* < 0.05, using the Mann–Whitney test wild type mice fed with high-saturated fat, high-fructose high-cholesterol diet versus high human soluble endoglin mice fed with high-saturated fat, high-fructose, high-cholesterol diet (WT_FFC_–hsEng_FFC_).

To set hsEng positive mice, the levels of hsEng present in the plasma of transgenic mice were set as an average 1472 ng/mL (with all mice expressing hsEng levels above the 1000 ng/mL threshold). Wild-type (WT) littermates presented undetectable hsEng levels in plasma (Figure 2A). 

**Figure 3:**
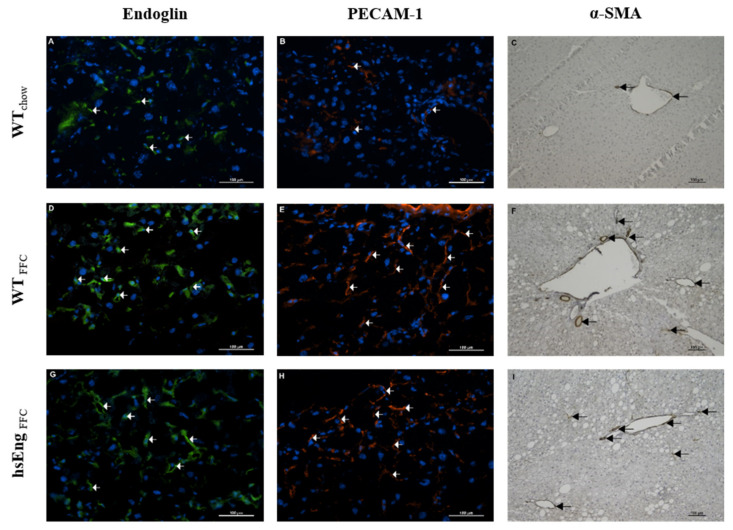
Representative pictures of immunohistochemical staining for Eng, PECAM-1, and anti-α-smooth muscle actin (α-SMA) in mice liver. Eng expression (green) is marked by arrows (white) in WT_chow_ (**A**), WT_FFC_ (**D**) and hsEng_FFC_ (**G**) mice. PECAM-1 expression (red) is marked by arrows (white) in WT_chow_ (**B**), WT_FFC_ (**E**) and hsEng_FFC_ (**H**) mice. Nuclei staining in blue. α-smooth muscle actin (brown) is marked by arrows (black) in WT_chow_ (**C**), WT_FFC_ (**F**) and hsEng_FFC_ (**I**) mice. Scale bar 100 µm.

Mice fed with an FFC diet showed increased levels of mouse soluble endoglin (msEng) by 42% (Figure 2B), matched to increased MMP14 protein expression by 70% (Figure 2C), the protease responsible for the Eng shedding into sEng. Additionally, immunohistochemical analysis showed increased endoglin expression in both FFC diet-fed mice (Figure 3D,G) with a similar staining pattern to endothelial cell marker Platelet Cell Adhesion Molecule-1 (PECAM-1) (Figure 3E,H) when compared to chow diet-fed mice (Figure 3A,B), suggesting increased expression of endoglin by the endothelium of sinusoids in the liver. Furthermore, we found mildly increased expression of anti-α smooth muscle actin (a marker of activated hepatic stellate cells and myofibroblasts [20]) in both FFC diet-fed mice (Figure 3F,I) compared to chow diet-fed mice (Figure 3C).

Moreover, an FFC diet significantly increased mRNA expression of Klf6, the transcription factor regulating the expression of Eng, by 166% (Figure 2D), and Eng protein levels in the liver by 42% (Figure 2E).

Although an FFC diet affected the mouse Eng and msEng levels, the presence of high levels of hsEng did not significantly modulate mouse Eng protein levels in the liver (Figure 2E) and msEng levels in plasma (Figure 2B). The immunohistochemical analysis did not reveal any visible differences in Eng, PECAM-1, and anti-α smooth muscle actin staining with respect hsEng levels (Figure 3G–I). Additionally, the presence of hsEng did not significantly affect *Klf6* mRNA expression (Figure 2D), and MMP14 protein expression (Figure 2C) in the liver.

### 2.3. High Soluble Endoglin Levels Increased Cholesterol and TAG in NASH Liver

**Figure 4:**
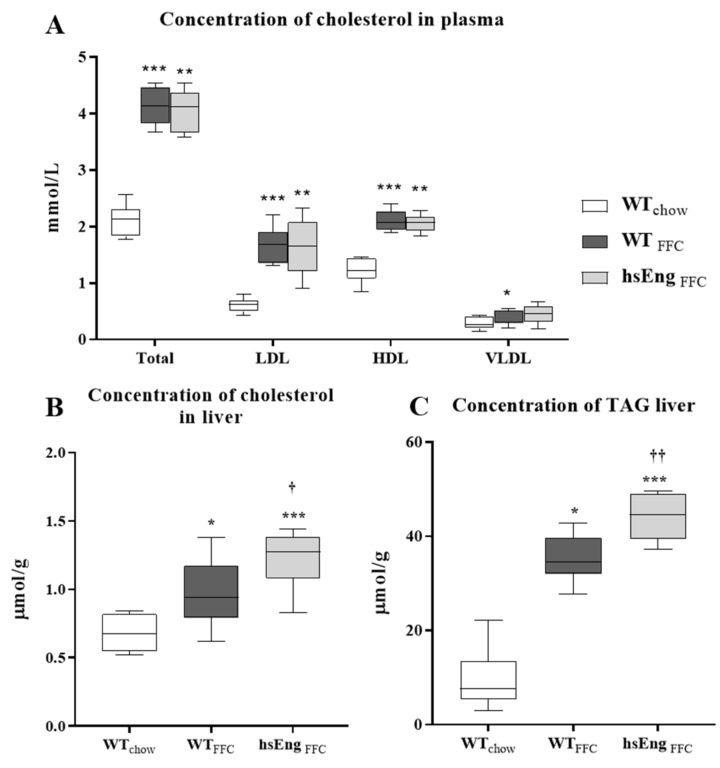
Cholesterol levels in blood and cholesterol and TAG concentration in mice liver. Cholesterol concentration in plasma (**A**). The concentration of cholesterol (**B**) and TAG (**C**) in the liver. The data are presented as median with box and whiskers representing the interquartile range and 5th–95th percentiles (*n* = 8). * *p* < 0.05, ** *p* < 0.01, *** *p* < 0.001, using the Kruskal–Wallis test; † *p* < 0.05, †† *p* < 0.01, using the Mann–Whitney test (WT_FFC_–hsEng_FFC_).

An FFC diet induced statistically significant hypercholesterolemia (Figure 4A) in both WT and hsEng mice by increasing cholesterol plasma levels (~97%) compared to the control animals. The increase was mostly related to increased LDL cholesterol levels (~53%) rather than high density lipoproteins (HDL) cholesterol levels (~43%), and a mild increase in VLDL cholesterol. Additionally, mice fed with an FFC diet showed increased cholesterol content in the liver (Figure 4B), in both WT (by 41%) and hsEng (by 80%), together with significantly increased levels of TAG content (Figure 4C) in both WT (by 266%) and hsEng (by 360%), when compared to control animals.

Despite the absence of a significant effect of hsEng on plasma levels of cholesterol, significantly increased levels of liver cholesterol (by 28%) and TAG content (by 25%) were found in the FFC diet-fed hsEng animals when compared to the FFC diet-fed WT mice (Figure 4B,C respectively).

### 2.4. FFC Diet Induces Steatosis and Promotes Fibrosis and Oxidative Stress in Liver

**Figure 5:**
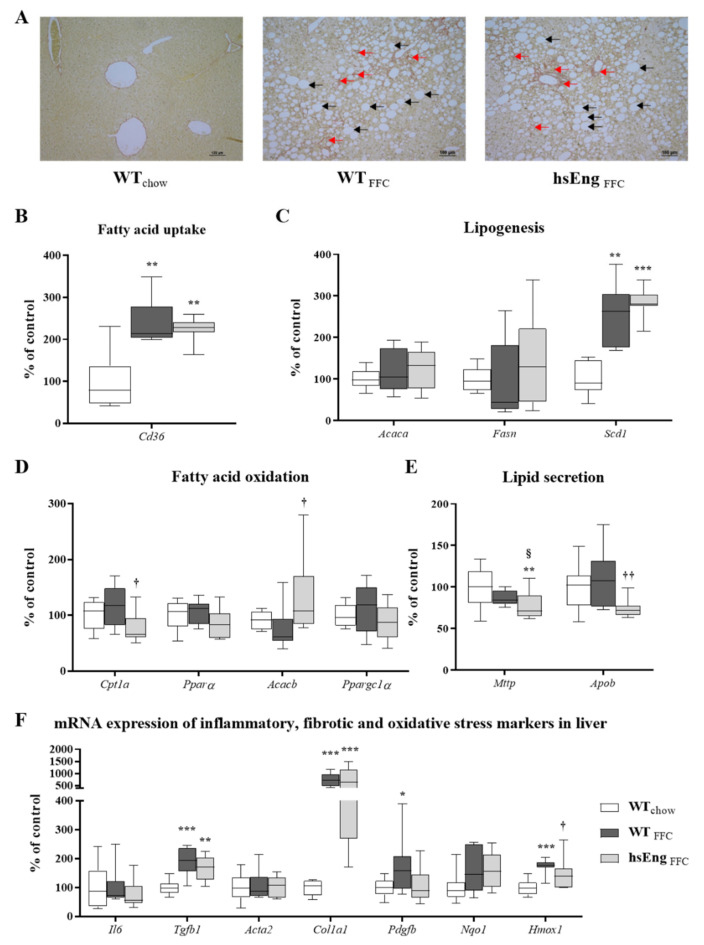
Evaluation of steatosis, fibrosis, inflammation, and oxidative stress in the liver. Sirius red-stained liver sections [macrovesicular steatosis–black arrows and mild fibrosis–red arrows] (**A**). mRNA expression of genes involved in FA uptake (*Cd36)* (**B**), lipogenesis (*Acaca, Fasn, Scd1)* (**C**) and oxidation (*Cpt1a, Pparα, Acacb, Ppargc1α*) (**D**). mRNA expression of genes involved in lipid secretion (*Mttp, Apob*) (**E**). Gene expression of inflammatory (*Il6)*, fibrogenic (*Tgf-β1* *, Acta2, Col1a1, Pdgfb*) and oxidative stress (*Nqo1, Hmox1)* markers (**F**). The data are presented as median with box and whiskers representing the interquartile range and 5th–95th percentiles (*n* = 8). * *p* < 0.05, ** *p* < 0.01, *** *p* < 0.001, using the Kruskal–Wallis test; † *p* < 0.05, †† *p* < 0.01 using the Mann–Whitney test (WT_FFC_–hsEng_FFC_); § *p* = 0.065 using the Mann–Whitney test (WT_chow_–WT_FFC_).

A NASH diagnosis was confirmed by histologic evaluation of the liver section, where mice fed with an FFC diet showed significant steatosis (macrovesicular steatosis), and mild induction of the fibrotic process (Figure 5A). We did not observe any difference in steatosis and fibrosis in FFC diet-fed mice regardless of the sEng levels (Figure 5A). 

To explore the mechanisms underlying the lipid accumulation and hepatic steatosis, we assessed changes in mRNA expression of crucial genes related to lipid metabolism, focusing on fatty acids (FA) uptake (*Cd36)* (Figure 5B), lipogenesis (*Acaca, Fasn, Scd1)* (Figure 5C), FA oxidation (*Cpt1a, Pparα, Acacb, Ppargc1α)* (Figure 5D), and lipid secretion (*Mttp, Apob)* (Figure 5E) in mice liver. qRT-PCR demonstrated that the expression of *Cd36* (cluster differentiation protein 36) (Figure 5B), the gene that mediates FA uptake, was elevated in the both FFC diet groups (by ~120%), as was the expression of stearoyl-CoA desaturase 1 (*Scd1)* (by ~155%), the gene involved in lipogenesis. Conversely, no significant expression changes in both acetyl-CoA carboxylase alpha (*Acaca*) and FA synthase (*Fasn*) were detected (Figure 5C). Additionally, genes associated with FA oxidation such as carnitine palmitoyl-transferase 1a (*Cpt1a*), peroxisome proliferator activated receptor alpha (*Pparα*), acetyl-CoA carboxylase beta (*Acacb*), peroxisome proliferator activated receptor gamma coactivator 1 alpha (*Ppargc1α*), were not affected by an FFC diet in WT animals (Figure 5D). Similarly, genes related to lipid secretion such as microsomal triglyceride transfer protein (*Mttp*) and apolipoprotein B (*Apob*) were not affected by an FFC diet in WT animals (Figure 5E). 

High levels of hsEng did not significantly affect FA uptake via *Cd36* (Figure 5B) nor lipogenesis via *Acaca, Fasn* nor *Scd1* (Figure 5C). Despite no significant change in *Pparα* and *Ppargc1α* (Figure 5D), increased levels of sEng significantly reduced the expression of *Cpt1a* by 40% and upregulated the expression of *Acacb* by 59% (Figure 5D), which indicates a pathway for reduced oxidation of FA. Additionally, hsEng significantly modulated lipid secretion (Figure 5E) by reducing the expression of *Apob* (~35%) and *Mttp* in hsEng mice, when compared to FFC diet animals. These findings of reduced oxidation of FA in association with reduced lipid secretion may explain the increased TAG content in the liver.

Indeed, an FFC diet significantly induced mRNA expression of fibrotic markers *Tgf-β1* (increased by more than 67%), *Col1a1* (increased by 635%), *Pdgfβ*, and oxidative stress marker *Hmox1* (Figure 5F). Quite the opposite, an FFC diet resulted in no significant change in the inflammatory marker *Il6,* fibrosis *Acta2,* or oxidative stress marker *Nqo1* (Figure 5F). Together, these data indicate the development of NASH with fibrosis by experimental diet.

Increased levels of hsEng did not significantly modulate any inflammation marker (*Il6)*, fibrosis (*Tgf-β1, Acat2, Col1a1, Pdgfβ*), nor oxidative stress (*Nqo1)*, besides *Hmox1* (Figure 5F).

### 2.5. High sEng Levels do not Modulate Hepatic Cholesterol Metabolism in FFC Diet-Induced Mouse Model

**Figure 6:**
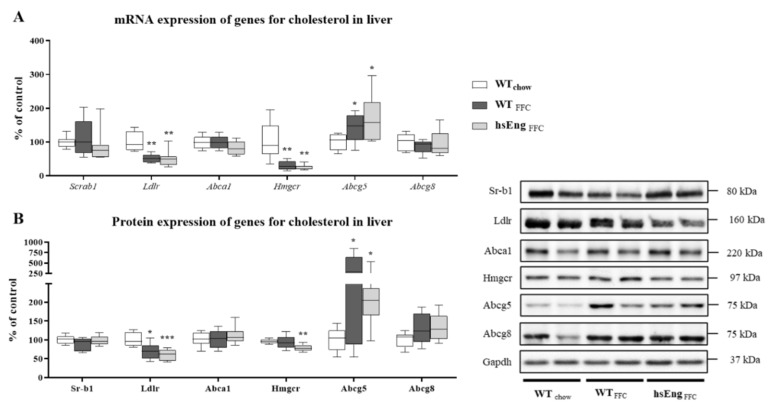
Cholesterol metabolism in the liver in WT and hsEng mice. mRNA (**A**) and protein (**B**) expression of intake transporter for HDL cholesterol (*Scrab1*/Sr-b1), LDL influx transporter (Ldlr), efflux transporter of cholesterol to plasma (Abca1), the rate-limiting enzyme for cholesterol de novo synthesis (Hmgcr), and transporters for the elimination of cholesterol into bile (Abcg5/Abcg8) in the liver. The data are presented as median with box and whiskers representing the interquartile range and 5th–95th percentiles (*n* = 8). * *p* < 0.05, ** *p* < 0.01, *** *p* < 0.001, using the Kruskal–Wallis test.

An FFC diet induced hypercholesterolemia and high levels of hsEng resulted in greater deposition of cholesterol in the liver. To elucidate these changes, an analysis of the cholesterol transporters and enzymes for de novo synthesis in the liver was performed. An FFC diet significantly reduced mRNA expression of the LDL receptor (Ldlr) (by 49%) (Figure 6A) and the protein level (by 30%) (Figure 6B). This may justify the increased levels of cholesterol in plasma (by its reduced intake to the liver), despite no significant change of the HDL intake transporter (*Scrab1*/Sr-b1) in the FFC diet groups. Additionally, an FFC diet downregulated both mRNA expression and protein levels (Figure 6A,B) of Hmgcr (the enzyme responsible for de novo synthesis of cholesterol) and increased Abcg5, despite no change in Abcg8 (efflux transporters of cholesterol to bile). Notwithstanding the attempt to reduce liver cholesterol content by reducing its de novo synthesis and increase elimination to bile, an FFC diet did not significantly affect the Adenosine triphosphate (ATP)-binding cassette transporter A1(Abca1) expression (Figure 6A,B), an efflux transporter of cholesterol to plasma.

High levels of hsEng did not modulate the cholesterol metabolism in the liver since no significant changes in *Scrab*/Sr-b1, Ldlr, Abca1, Hmgcr, nor Abcg5/8, were observed when compared to WT animals (Figure 6A,B).

### 2.6. sEng and FFC Diet Modulates Bile Acid Metabolism in the Liver

**Figure 7:**
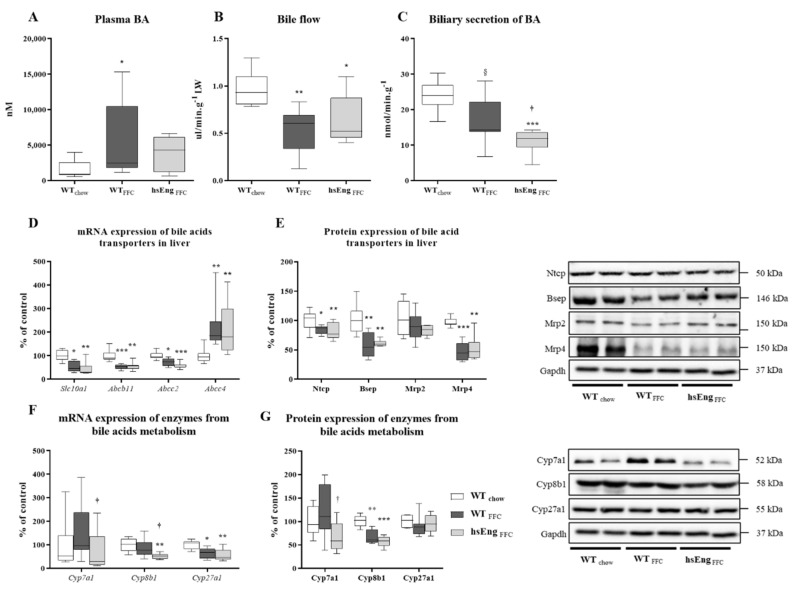
Bile acid metabolism in the liver in WT and hsEng mice. Plasma concentration of BA (**A**). Bile flow rate (**B**). Biliary elimination of BA (**C**). mRNA (**D**) and protein (**E**) expression of transporters involved in BA uptake to the liver (*Slc10a1*/Ntcp), bile production (*Abcb11*/Bsep, *Abcc2*/Mrp2), and BA efflux to plasma (*Abcc4*/Mrp4). mRNA (**F**) and protein (**G**) expression of liver enzymes involved in BA synthesis from cholesterol (Cyp7a1, Cyp8b1, Cyp27a1). The data are presented as median with box and whiskers representing the interquartile range and 5th–95th percentiles (*n* = 8). * *p* < 0.05, ** *p* < 0.01, *** *p* < 0.001, using the Kruskal–Wallis test; † *p* < 0.05 using the Mann–Whitney test (WT_FFC_–hsEng_FFC_); § *p* = 0.053 using the Mann–Whitney test (WT_chow_–WT_FFC_).

To further characterize the accumulation of cholesterol in hsEng mice, we analyzed the biochemistry of BA. An FFC diet significantly increased the plasma concentration of BA (by 250%) (Figure 7A). When assessing mRNA expression (Figure 7D) and protein levels (Figure 7E) of BA transporters in liver, an FFC diet significantly reduced BA uptake to the liver from plasma via reduced *Slc10a1*/Ntcp (a sodium-dependent uptake transporter expressed on the basolateral membrane of hepatocytes responsible for the uptake of BA from plasma), mRNA expression (by ~50%), and protein levels (by 30%), which reflected an increased plasma concentration of BA in FFC diet-fed mice. Surprisingly, the Mrp4 protein level (an ATP-dependent unidirectional efflux transporter of BA to plasma) was decreased by an FFC diet when compared to its mRNA expression (Figure 7D,E). 

Conversely, an FFC diet reduced the bile flow rate by 45% (cholestatic effect) (Figure 7B) and biliary secretion of BA by 30% (Figure 7C) when compared to control animals. Consistent with observed mRNA expression patterns, the protein levels of Bsep (export transporter of BA in the canalicular membrane) (Figure 7E), were markedly downregulated in both FFC diet-fed animals (by 48% and 43%, respectively), which may explain the reduction of the bile flow rate by the decrease of bile acid-dependent bile flow (biliary secretion of BA) (Figure 7C). Regarding the bile acid independent bile flow, no change in Mrp2 protein levels (a canalicular transporter of glutathione) was observed, despite its downregulation in mRNA expression (Figure 7D). 

Regarding the liver enzymes responsible for BA synthesis from cholesterol, no significant change was observed for Cyp7a1 expression between control and WT_FFC_ mice (Figure 7F,G). Despite no significant change in mRNA expression (Figure 7F), the Cyp8b1 protein level (Figure 7G) was significantly reduced. Alternatively, reduced mRNA expression of *Cyp27a1* was not confirmed at the protein level (Figure 7F,G).

Increased levels of hsEng significantly reduced biliary secretion of BA when compared with the FFC diet WT group (Figure 7C). This reduction was consistent with reduced conversion of cholesterol into BA, via reduced mRNA expression and protein levels of Cyp7a1, the rate-limiting enzyme for the neutral pathway of BA synthesis (Figure 7F,G). Notably, hsEng did not significantly affect plasma concentration of BA (Figure 7A), the bile flow rate (Figure 7B), nor the modulated BA metabolism in the liver via a significant change in expression of transporters (Ntcp, Bsep, Mrp2 and Mrp4) (Figure 7D,E), nor expression of enzymes (Cyp8b1 and Cyp27a1) (Figure 7F,G) when compared to WT mice. Taken together, these results indicate that hsEng increased liver accumulation of cholesterol in the FFC diet fed mice by its reduced metabolism of BA.

## 3. Discussion and Conclusions

Eng expression and the presence of sEng in the circulation was discussed in relation to many pathological conditions, including various metabolic disorders such as hypercholesterolemia, atherosclerosis, type 2 diabetes mellitus, and liver fibrosis [16,17]. However, detailed experimental data on Eng and sEng effects during NASH are missing.

The main and novel findings of this study are related to the fact that Eng and sEng play a role in the pathology of experimental NASH in mice. First, NASH increased liver Eng expression, which resulted in simultaneously increased levels of sEng in plasma. Second, the presence of additional high levels of sEng in blood in transgenic mice resulted in an aggravation of already developed NASH by increasing the liver content of TAG and cholesterol and reduced biliary secretion of BA. 

During this study, the successful development of NASH by feeding an FFC diet was indeed confirmed by histological and biochemical analysis as described previously [25,37]. Moreover, an FFC diet increased liver Eng protein together with *Klf6*, the transcription factor responsible for upregulation of Eng expression under the vascular injury [38]. Overexpression of *Klf6* was demonstrated in the liver alteration/injury in NAFLD as well [39]. An FFC diet also significantly increased the mRNA expression of profibrotic biomarkers *Tgf-β1*, *Pdgfβ*, and *Col1a1* in the liver [25]. Indeed, it was demonstrated that fibrosis is the result of abnormal synthesis and the accumulation of type I collagen in the extracellular matrix produced by activated stellate cells in the damaged liver [40,41]. Importantly, the up-regulation of Eng expression was observed during hepatic stellate cell activation and their transdifferentiation to myofibroblast-like cells in cell cultures and both mice and rat experimental models of liver fibrosis such as CCl_4_ application and bile duct ligation [2,42].

Moreover, increased expression of Eng was reported in patients with the hepatitis C virus infection with advanced fibrosis compared to patients with early fibrosis and a healthy liver [20]. These results are consistent with enhanced Eng-*Tgf-β1-Col1a1* signaling in our mice with NASH. Thus, we propose that Eng participates in the regulation of fibrotic processes during NASH. Conversely, it is of interest to mention that Alsamman et al. 2018 suggested Eng as a protective factor against fibrotic injury because deficiency of Eng significantly aggravated CCl_4_ and bile duct ligation-induced liver fibrosis by modulating Tgf-β/Smad signaling [19]. Interestingly, Eng expression in the liver was similar to PECAM-1 expression, which is a biomarker of endothelial cells. Both proteins’ expression was induced by an FFC diet, suggesting possible stimulation of angiogenesis, which might be considered a protective mechanism in the NASH-damaged liver [43]. Then again, we found only weak co-expression of Eng with α smooth muscle actin, which was shown to be a biomarker of activated hepatic stellate cells [20]. Thus, the question whether increased Eng expression supports pathological fibrogenesis or stimulates protective angiogenesis remains to be elucidated. 

During our study, NASH increased both *Klf6* and MMP14 expression in the liver and levels of mouse sEng in plasma, suggesting that increased release of sEng is related to pathological changes in the liver induced by the FFC diet. This is in line with some previous studies showing increased circulating levels of sEng in the serum of patients with hepatic fibrosis featuring chronic hepatitis C virus infection (HCV) [20], patients with biliary atresia [21], cystic fibrosis-associated liver disease (CFLD) [44], and patients with liver cirrhosis and carcinoma [45]. Moreover, it was shown that sEng levels were increased during hypercholesterolemia [11,46]. Interestingly, several studies have shown that membrane MMP14 cleaves Eng in a trophoblast-like cell line JAr choriocarcinoma cell line (JAR) [10], Human umbilical vein endothelial cells (HUVECs) [9], breast cancer cell line Michigan Cancer Foundation-7 (MCF-7) [47], and releases sEng into cell culture media. Besides, *Klf6* was shown to upregulate MMP14 expression, suggesting both *Klf6* and MMP-14 might be involved in the formation of sEng during NASH development [38]. 

Therefore, NASH development, together with hypercholesterolemia, resulted in the elevation of plasma sEng in both our experimental groups, suggesting that sEng might be a circulating biomarker of NASH. 

Transgenic mice with high levels of hsEng represent an interesting tool to study its effects in various organs and pathological conditions, including preeclampsia, endothelial dysfunction, and arterial hypertension [34,46,48,49]. Thus, we aimed to evaluate the potentially harmful effects of sEng in NASH pathophysiology. 

First, we aimed to explore the mechanisms underlying increased TAG levels in high human soluble endoglin high-saturated fat, high-fructose high-cholesterol fed (hsEng_FFC_) mice. We assessed changes in mRNA expression of crucial genes related to lipid metabolism (fatty acid uptake, lipogenesis, fatty acid oxidation, and lipid secretion) in mice livers. Hepatic steatosis is characterized by excessive deposition of triglyceride-rich lipid droplets in the liver that occurs when the rate of import or synthesis of fatty acids (FA) by hepatocytes exceeds the rate of catabolism or export [50,51]. The presence of high sEng significantly increased the expression of *Acacb*, together with significant downregulation of *Cpt1a*. Acetyl Coenzyme A Carboxylase (ACC) plays a pivotal role in FA liver turnover by catalyzing the interconversion of acetyl-CoA and malonyl-CoA. There are two isoforms of Acc, Acaca and Acacb, which have distinct roles in FA synthesis and oxidation, respectively [52,53]. Acacb catalyzes carboxylation of acetyl-CoA to malonyl-CoA. Increased levels of malonyl-CoA are known to inhibit Cpt1 (carnitine palmitoyltransferase 1), which is responsible for the shuttle of FA into mitochondria for β-oxidation [52,53,54]. Thus, we propose that the inhibition of Cpt1 and β-oxidation in hsEng_FFC_ mice resulted in increased availability of FA for TAG synthesis and their liver storage [55].

Moreover, mice with high sEng levels showed significantly reduced *Apob* and *Mttp* (with borderline statistical significance) expression in the liver when compared to WT_FFC_ mice. Synthesis of TAG in the liver is usually matched with their secretion in the form of very low density lipoproteins (VLDL), except in aberrant situations leading to fatty livers [56]. Mttp plays an essential role in the transfer of lipids to apolipoprotein B during the early stages of lipoprotein assembly, therefore, being essential for the assembly and secretion of apoprotein β-containing lipoproteins [57,58]. Thus, the inhibition of *Apob* and *Mttp* in hsEng_FFC_ mice demonstrates another potential mechanism responsible for increased intracellular TAG accumulation in the liver [59,60]. Taken together, reduced β-oxidation of FA and altered formation and secretion of lipoproteins may explain the increased deposition of TAG in the liver observed in hsEng_FFC_ mice.

The previously described association between hypercholesterolemia and sEng levels [46] led us to the evaluation of liver cholesterol metabolism. Mice with high plasma levels of hsEng showed further increased accumulation of cholesterol in the liver when compared to WT_FFC_ mice. Interestingly, neither of the analyzed molecules required for liver cholesterol synthesis and transport showed a significant change in the presence of high levels of sEng, suggesting the existence of another mechanism. This is partially in contrast to our previous paper, where high levels of sEng in chow diet-fed animals resulted in elevated cholesterol content in the liver via activation of the Srebp2-Ldlr axis [34]. Such a discrepancy suggests that lipid overload and the complex pathology of NASH markedly modulates the regulatory role of sEng, and different liver responses may be expected in situations with increased sEng and intact livers. 

Indeed, the concentration of cholesterol also is regulated by its hepatic conversion into BA [61]. Interestingly, we demonstrated in our previous study that sEng upregulates Cyp7a1 in transgenic mice on a chow diet [62]. The same strain of hsEng mice on an FFC diet in the present study showed downregulated Cyp7a1 in comparison with the WT_FFC_ group. This discrepancy may correspond with NASH-mediated activation of several pathways [63] that suppress Cyp7a1, such as Tgf-β, also seen in our study. Although the exact regulatory pathway involved in this sEng effect requires further clarification, the absence of other obvious changes in crucial cholesterol homeostatic pathways suggests that downregulation of Cyp7a1-mediated BA synthesis is the principal mechanism behind increased cholesterol liver accumulation in sEng mice with NASH.

Most importantly, mice with high sEng levels showed reduced BA biliary secretion without significant change in the expression of crucial transporters, including bile salt export pump (Bsep). Thus, we propose that Cyp7a1-mediated BA synthesis reduced by sEng might be the principal mechanism of reduced BA biliary secretion, which results in increased liver cholesterol content in hsEng mice.

**Figure 8:**
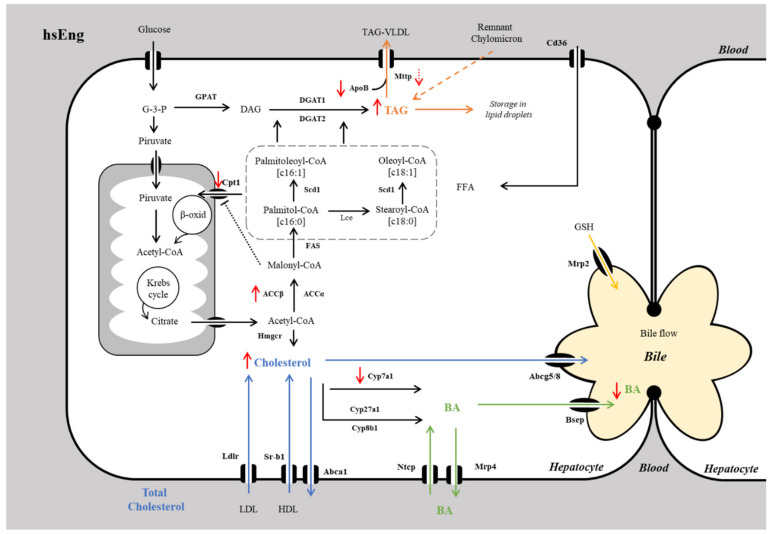
Schematic representation of hsEng effects in lipid, cholesterol, and BA metabolic pathways in the NASH diet-induced mouse model. Increased hepatic TAG content may result from decreased beta-oxidation of fatty acids (via Accb and Cpt1) and its reduced efflux to plasma (by Apob and Mttp). Reduced conversion of cholesterol into BA (via Cyp7a1) may be the reason for increased cholesterol accumulation and consequent lower biliary elimination of BA in a NASH liver. Full red arrow—statistically significant. Dotted red arrow—borderline significance. Blue arrows—circulation of cholesterol. Green arrows—circulation of bile acids. Yellow arrow—efflux of glutathione. Orange arrows—circulation of TAG. Black arrows metabolic pathways. Abca1, ATP-binding cassette transporter; Abcg5/8, Dimer of ATP-binding cassette subfamily G member 5 and member 8; ACC, acetyl-CoA carboxylase; Apob, Apolipoprotein B; β-oxid, beta-oxidation; BA, bile acids; Bsep, Bile salt export pump; Cd36, cluster of differentiation 36; DAG, Diacylglycerol; DGAT, Diglyceride acyltransferase; FAS, Fatty acid synthase; FFA, free fatty acids; G-3-P, Glyceraldehyde-3-phosphate; GPAT, Glycerol-3-phosphate acyltransferase; GSH, Glutathione; HDL, high-density lipoproteins; Hmgcr, 3-Hydroxy-3-methylglutaryl-CoA reductase; hsEng, Human soluble endoglin; Lce, long chain fatty acyl elongase; LDL, low-density lipoproteins; Ldlr, LDL receptor; Mrp2, Multidrug resistance-associated protein 2; Mrp4, Multidrug resistance-associated protein 4; Mttp, Microsomal triglyceride transfer protein; Ntcp, sodium/taurocholate cotransport polypeptide; Scd1, Stearoyl-CoA desaturase-1; Sr-b1, Scavenger receptor class B type 1; TAG, Triacylglycerol.

To summarize the results, we show a graphic explanation in Figure 8.

Thus, we demonstrate, for the first time, increased circulating sEng levels in NASH. This increment might be related to the increased expression of Eng in the liver and increased levels of MMP14, the protease responsible for the shedding of Eng, and the release of its soluble form into circulation. Moreover, mice with high levels of sEng showed the increased hepatic deposition of cholesterol via downregulation of Cyp7a1, reducing its conversion into BA. Regarding mice with high sEng levels, redirection of the metabolism of TAG to its accumulation in the liver via reduced TAG elimination by β-oxidation combined with reduced hepatic efflux was shown as well. 

There are some limitations of the study. We propose the association of Eng/sEng with NASH, however, we are unable to elucidate the exact molecular mechanism that results in these changes. Indeed, the only difference between WTFFC and hsEngFFC mice is the presence of high levels of human soluble endoglin in hsEngFFC mice. Other biochemical parameters (lipid levels in the blood), levels of mouse soluble endoglin, and endoglin liver expression did not significantly differ between WTFFC and hsEngFFC in this study, which suggests an sEng role in NASH aggravation. Moreover, we cannot use knock-out endoglin mice for an in vivo study because mice lacking both copies of the endoglin gene die at gestational day 10.0–10.5 due to defects in the vessel and heart development [64]. Since we do not know if and how soluble endoglin affects directly described metabolic changes, prospective studies are necessary to obtain mechanistic insights into soluble endoglin effects during NASH. 

To conclude, we propose that sEng might be a biomarker of NASH development and the presence of high levels of sEng might co-participate in NASH aggravation by impairing the essential defensive mechanism protecting a NASH liver against excessive TAG and cholesterol accumulation, suggesting the importance of high sEng levels in patients prone to develop NASH.

## 4. Materials and Methods

### 4.1. Animals and Experimental Design

Three-month-old transgenic male mice overexpressing human sEng on the CBAxC57BL/6J background (hsEng), generated at the Genetically Modified Organisms Generation Unit (University of Salamanca, Spain), as previously described [10], and their wild type littermates (WT) (*n* = 8 in each group) were fed for 6 months either with a chow diet (PicoLab Rodent Diet20 and water ad libitum) or a Western-type diet (AIN-76A WD, TestDiet) containing 45% kcal fat (milk fat), 0.2% cholesterol, 15.4% protein (casein), and 44.5% carbohydrate (sucrose) with additional glucose (18.1 g/L) and fructose (24 g/L) added in drinking water. The former diet was previously termed as a high-saturated fat, high-fructose, and high-cholesterol (FFC) diet [25]. 

The animals were housed under a 12-h light cycle and constant temperature (22 ± 1 °C) and had free access to a respective drink and pellet diet. Individual mouse body weight was registered weekly from week 0 up to week 24 of feeding with the chow or FFC diet. All animals received care according to the guidelines set by the Animal-Welfare Body of the Faculty of Pharmacy in Hradec Kralove, Charles University (Reg. Number: 21558/2013–2, date May 2^nd^, 2019), and the Bioethics Committee of the University of Salamanca (Permit Number: 006–201400038812, date October 22^nd^, 2014). This mouse model of diet-induced NASH resulted in NASH with hepatocyte ballooning and fibrosis displaying high fidelity to human NASH in C57BL6 mice, as previously described [25,35,36].

### 4.2. In Vivo Clearance Study

After overnight fasting, mice underwent an in vivo clearance study. Under anesthesia induced by isoflurane (Vetflurane^®^, Virbac, Kolding, Denmark), followed by intraperitoneal administration of pentobarbital (50 mg/kg), the gall bladder was cannulated under the stereomicroscope for bile collection in pre-weighed tubes for 45 min. The body temperature of the animals was preserved at 37 °C by keeping the animals on a heated platform. Blood samples were taken from the vena cava inferior at the end of the experiment, and mice were sacrificed by anesthetic overdose. Harvested livers were immediately frozen in liquid nitrogen. Plasma samples were obtained from the whole blood by centrifugation at 2000× *g* for 5 min at 4 °C. Samples were stored at −80 °C until analysis.

### 4.3. Analytical Methods

Serum lipoprotein fractions were prepared using sodium chloride density gradient ultracentrifugation (Optima MAX-XP Ultracentrifuge, Beckman–Coulter, Palo Alto, CA, U.S.A.). The lipoprotein fractions were distinguished in the following density ranges: VLDL < 1.006 g/mL, LDL 1.006–1.063 g/mL, HDL > 1.063 g/mL. Total cholesterol and triacylglycerol (TAG) concentrations and lipoprotein fraction concentrations of cholesterol and TAG were measured enzymatically by conventional enzymatic diagnostic kits (Erba Lachema s.r.o., Brno, Czech Republic) and spectrophotometric analysis (cholesterol at 510 nm, TAG at 540 nm; ULTROSPECT III, Pharmacia LKB Biotechnology, Uppsala, Sweden). 

To determine cholesterol and TAG in the liver; tissues were homogenized and extracted for 16 h in Folch’s solution (chloroform:methanol mixture [2:1]), after which 2% KH_2_PO_4_ was added, and the solution was centrifuged. The organic phase was removed and evaporated under N_2_. Total cholesterol and TAG content were determined by the enzymatic assay as mentioned above.

Liver enzymes in plasma were detected using a commercial Preventive Care Profile Plus test and Vetscan 2 device (Abaxis, Germany).

### 4.4. Analysis of Bile Acids in Plasma and Bile 

Analysis of endogenous BA content in plasma and bile was performed using liquid chromatography coupled with mass spectrometry (LC–MS) as described previously [34]. 

### 4.5. Quantitative Real-Time RT-PCR

Gene expression was performed by reverse transcription-polymerase chain reaction (qRT-PCR) in QuantStudio 6 Flex Real-Time PCR Software (Applied Biosystems, Thermo Fisher Scientific, Waltham, MA USA) as previously described [65]. Primers used for analysis are specified in Supplementary Table S1. The Glyceraldehyde 3-phosphate dehydrogenase (*Gapdh*) gene was used as a reference for normalizing data. 

### 4.6. Western Blot Analysis

The total fraction and crude plasma membrane fraction were prepared from mice liver homogenates, as described previously [66]. Proteins (25 µg) were separated by SDS–PAGE, transferred to the PVDF membrane (Millipore, New York, NY, USA), and incubated with appropriate antibodies (Supplementary Table S2). The Chemiluminescence image of bands was captured using Evolution-capt software (Fusion Solo 6S Edge, Vilber Lourmat SAS, France) and quantified using ImageLab imaging software version 6.0.1 (Bio–Rad). The equal loading of proteins onto the gel was confirmed by the immunodetection of Gapdh. 

### 4.7. Histology and Immunohistochemical Analysis

Liver sections were fixed in 4% paraformaldehyde, embedded in paraffin, and serial cross-sections (7 µm) were cut and stained with Sirius Red (SR) to visualize steatosis and fibrosis.

Concerning immunohistochemical analysis, serial cross-sections (7 µm) were cut on a cryostat and placed on gelatin-coated slides. Five slides from each animal and from each group were taken for the analysis. Slides were incubated with either rat anti-mouse endoglin (1:50, Pharmingen) or rat anti-mouse PECAM-1 primary antibody (1:50, Pharmingen). After that, slides were incubated with secondary antibodies, either Fluorescein Rabbit Anti-Rat IgG Antibody (green, 1:100) for the detection of endoglin, or CY3 Goat Anti-Rabbit IgG Antibody (red, 1:100) for the detection of PECAM-1 (Vector Laboratories). Regarding nuclear counterstaining, the blue-fluorescent Hoechst nucleic acid stain (Invitrogen) was used. The Rabbit ImmPress® polymerized reporter enzyme staining system (Vector) was used for the detection of the rabbit anti-α smooth muscle actin (1:50, Abcam) primary antibody, and the reactivity was detected by means of diaminobenzidine tetrahydrochloride substrate (Dako) with hematoxylin counterstaining. The specificity of the immunostaining was assessed by staining with nonimmune isotype-matched immunoglobulins. Photo documentation and image digitizing from the microscope were performed using the Olympus AX 70, with a NIKON DS-Fi3, a high-definition color microscope camera, and image analysis software NIS (Laboratory Imaging, Czech Republic). 

### 4.8. Enzyme-Linked Immunosorbent Assay

Transgenic mice express variable levels of human soluble endoglin (hsEng) so, to achieve comparable results, Enzyme-Linked ImmunoSorbent Assay (ELISA) was performed to determine the concentration of hsEng present in mice plasma. Plasma was analyzed using the Human Endoglin/CD105 Quantikine ELISA kit (DNDG00, R&D Systems, Minneapolis, MN, USA) according to the manufacturers’ instructions, in duplicates. Transgenic mice (hsEng) were selected according plasma levels of hsEng above 1000 ng/mL threshold. Conversely, their wild-type (WT) littermates presented undetectable hsEng levels in plasma. 

To determine the plasma mouse sEng (msEng) levels, a Mouse Endoglin/CD105 Quantikine (MNDG00, R&D Systems, Minneapolis, MN, USA) ELISA Kit was used, according to the manufacturers’ instructions, in duplicates.

### 4.9. Statistical Analysis

All analyses were performed using GraphPad Prism 8.0 software (San Diego, CA, USA). The data are presented as medians with box and whiskers representing the interquartile range and 5th–95th percentiles, respectively, according to the data distribution. Comparisons between the FFC diet groups to the control were carried out using the Kruskal–Wallis test and direct comparisons between WT_FFC_–hsEng_FFC_ were carried out using the Mann–Whitney test to verify significance. A difference of *p* < 0.05 was considered statistically significant.

## Supplementary Materials

The following are available online at https://www.mdpi.com/1422-0067/21/23/9021/s1.
