# Supplementary material for: Soluble Endoglin as a Potential Biomarker of Nonalcoholic Steatohepatitis (NASH) Development, Participating in Aggravation of NASH-Related Changes in Mouse Liver
Source: Int J Mol Sci. 2020 Nov 27;21(23):9021. doi: 10.3390/ijms21239021 (PMC7731045; doi:10.3390/ijms21239021)
Supplement: Supplementary file 1 [file ijms-21-09021-s001.pdf]

**Table S1** Pre-designed TaqMan® Gene Expression Assay kits (Life Technologies) used for quantitative real-time RT-PCR

| Gene symbol | Transporter/Receptor | Life Technologies cat. number |
|---|---|---|
| *Klf6* | | Mm00516184_m1 |
| *Cd36* | | Mm00432403_m1 |
| *Acaca* | | Mm01304257_m1 |
| *Fasn* | | Mm00662319_m1 |
| *Scd1* | | Mm00772290_m1 |
| *Cpt1a* | | Mm01231183_m1 |
| *Pparα* | | Mm00440939_m1 |
| *Acacb* | | Mm01204671_m1 |
| *Ppargc1α* | | Mm01208835_m1 |
| *Mttp* | | Mm00435015_m1 |
| *Apob* | | Mm01545150_m1 |
| *IL-6* | | Mm00446190_m1 |
| *Tgf-β1* | | Mm01178820_m1 |
| *Acta2* | α-SMA | Mm01546133_m1 |
| *Col1a1* | | Mm00801666_g1 |
| *Pdgfb* | | Mm00440677_m1 |
| *Nqo1* | | Mm01253561_m1 |
| *Hmox1* | | Mm00516005_m1 |
| *Scrab1* | Sr-b1 | Mm00450234_m1 |

| | | |
|---|---|---|
| *Ldlr* | | Mm01177349_m1 |
| *Abca1* | | Mm00442646_m1 |
| *Hmgcr* | | Mm01282499_m1 |
| *Abcg5* | | Mm00446241_m1 |
| *Abcg8* | | Mm00445980_m1 |
| *Slc10a1* | Ntcp | Mm00441421_m1 |
| *Abcb11* | Bsep | Mm00445168_m1 |
| *Abcc2* | Mrp2 | Mm00496899_m1 |
| *Abcc4* | Mrp4 | Mm01226380_m1 |
| *Cyp7a1* | | Mm00484150_m1 |
| *Cyp8b1* | | Mm00501637_s1 |
| *Cyp27a1* | | Mm00470430_m1 |
| *Gapdh* | | Mm99999915_g1 |

**Table S2** Primary and secondary antibodies used in Western blot

| Protein | Source | Dilution | Secondary antibody dilution |
|---|---|---|---|
| Eng | Santa Cruz (sc19793) | 1:500 | 1:3000 |
| MMP14 | Abcam (ab51074) | 1:2000 | 1:4000 |
| Sr-b1 | Novus (NB400-104) | 1:2000 | 1:4000 |
| Ldlr | Thermo Fisher (PA5-20752) | 1:1000 | 1:2000 |
| Abca1 | Thermo Fisher (PA1-16789) | 1:500 | 1:2000 |
| Hmgcr | Thermo Fisher (PA5-37367) | 1:1000 | 1:2000 |
| Abcg5 | Thermo Fisher (PA5-69249) | 1:1000 | 1:2000 |
| Abcg8 | Thermo Fisher (PA1-16798) | 1:500 | 1:2000 |
| Ntcp | Santa Cruz (sc94845) | 1:3000 | 1:6000 |
| Bsep | Thermo Fisher (PA5-13105) | 1:1000 | 1:4000 |
| Mrp2 | Thermo Fisher (PA5-49997) | 1:500 | 1:1000 |
| Mrp4 | Cell Signaling (#12857S) | 1:2000 | 1:4000 |
| Cyp7a1 | Sigma (MABD42) | 1:2000 | 1:5000 |
| Cyp8b1 | Thermo Fisher (PA5-37088) | 1:1000 | 1:2000 |
| Cyp27a1 | Thermo Fisher (PA5-27946) | 1:1000 | 1:2000 |
| Gapdh | Cell Signaling (#2118) | 1:8000 | 1:10000 |
